# Comorbid Conditions in Chronic Obstructive Pulmonary Disease: Potential Therapeutic Targets for Unmet Needs

**DOI:** 10.3390/jcm9103078

**Published:** 2020-09-24

**Authors:** Kazuto Matsunaga, Misa Harada, Junki Suizu, Keiji Oishi, Maki Asami-Noyama, Tsunahiko Hirano

**Affiliations:** 1 Department of Respiratory Medicine and Infectious Disease, Graduate School of Medicine, Yamaguchi University, Ube 755-8505, Japan; hara-da@yamaguchi-u.ac.jp (M.H.); relativity.theory135@gmail.com (J.S.); noyamama@yamaguchi-u.ac.jp (M.A.-N.); tsuna@yamaguchi-u.ac.jp (T.H.); 2 Department of Medicine and Clinical Science, Graduate School of Medicine, Yamaguchi University, Ube 755-8505, Japan; ohishk@yamaguchi-u.ac.jp

**Keywords:** COPD, cardiovascular disease, bronchiectasis, frailty, asthma

## Abstract

The management of chronic obstructive pulmonary disease (COPD) has improved significantly due to advances in therapeutic agents, but it has also become apparent that there are issues that remain difficult to solve with the current treatment algorithm. COPD patients face a number of unmet needs concerning symptoms, exacerbations, and physical inactivity. There are various risk factors and triggers for these unmet needs, which can be roughly divided into two categories. One is the usual clinical characteristics for COPD patients, and the other is specific clinical characteristics in patients with comorbid conditions, such as asthma, cardiovascular disease, and bronchiectasis. These comorbidities, which are also associated with the diversity of COPD, can cause unmet needs resistance to usual care. However, treatable conditions that are not recognized as therapeutic targets may be latent in patients with COPD. We again realized that treatable traits should be assessed and treated as early as possible. In this article, we categorize potential therapeutic targets from the viewpoint of pulmonary and systemic comorbid conditions, and address recent data concerning the pathophysiological link with COPD and the impact of intervention on comorbid conditions in order to obtain evidence that could enable us to provide personalized COPD management.

## 1. Introduction

Chronic obstructive pulmonary disease (COPD) is a major health problem with increasing incidence and mortality worldwide [1,2]. The current mainstay of pharmacotherapy is bronchodilators, and recent advances in these agents have enabled further improvements in lung function and reductions in symptoms and exacerbations [3,4]. However, it has also become apparent that there are remaining issues that are difficult to resolve with the current treatment algorithm. COPD patients face a number of unmet needs such as symptoms, exacerbations, physical inactivity, and loss of social activities [5,6,7,8,9,10].

Most of the current treatment algorithms are based on the severity of symptoms and exacerbation history, and recommend the use of drugs other than inhalation medications when symptoms remain or exacerbations occur [3,11]. However, even a single exacerbation can accelerate the loss of lung function, inducing physical inactivity and increasing the risk of death [12,13]. A large-scale epidemiological study in Japan showed that dyspnea of the modified Medical Research Council (mMRC) grade 2 or more remains in almost one half of COPD patients managed by pulmonologists [14,15]. Given the burden of such unmet needs, it is very important to promote pre-emptive therapies that target treatable traits [5,6,7,8,9].

**Figure 1:**
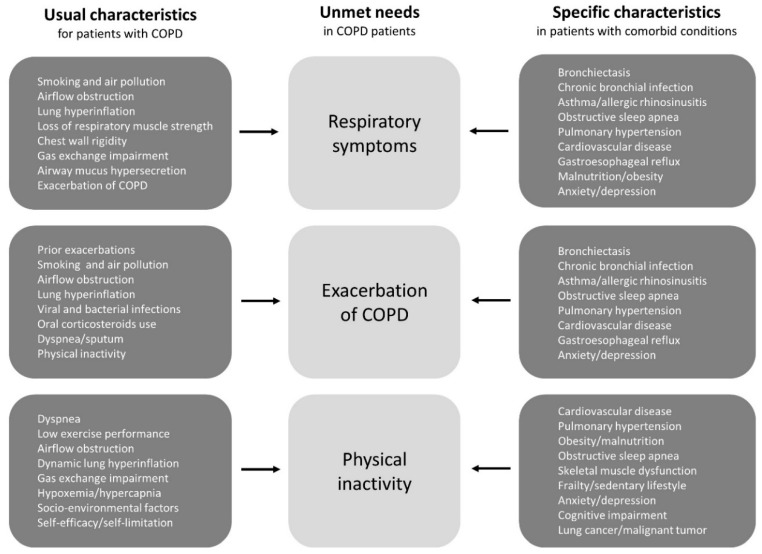
The risk factors and triggers for COPD patients’ unmet needs can be divided into two categories; one is the usual clinical characteristics of COPD patients and the other is the specific clinical characteristics of patients with comorbid conditions. Treatable conditions that are not recognized as therapeutic targets may be latent in patients with COPD. Abbreviations: COPD, chronic obstructive pulmonary disease.

There are various risk factors and triggers for patients’ unmet needs, while the factors can be roughly divided into two categories (Figure 1). One is the usual characteristics of COPD patients, such as smoking and airflow obstruction. As a standard approach, it goes without saying that smoking cessation, bronchodilator, pulmonary rehabilitation, and vaccination are established treatments, and they should be included under general recommendations for all patients [3,11]. The other includes specific characteristics in patients with comorbid conditions, such as asthma, bronchiectasis, and cardiovascular disease [9]. These comorbidities, which are also associated with the diversity of COPD [5,6,7,8,9], can cause patients’ unmet needs to resist usual care. However, treatable conditions that are not recognized as therapeutic targets may be latent in patients with COPD. However, there is accumulating evidence indicating that some clinical features and specific biomarkers can be used to identify patients who should be given second controllers, such as inhaled corticosteroids (ICS) [16,17,18], selective β1-blockers [19,20,21], and macrolides [22,23,24]. We again realized that treatable traits should be assessed and treated as early as possible [9].

In this article, we categorize potential therapeutic targets based on pulmonary and systemic comorbid conditions and address the data of pathophysiological relationships with COPD and the impact of intervention for these comorbid conditions to obtain evidence that will enable the development of personalized COPD management.

## 2. Bronchiectasis

Microorganisms are frequently observed in the airways of COPD patients, both in stable state and during exacerbations. The isolation of potentially pathogenic microorganisms (PPM) such as *Hemophilus influenzae*, *Streptococcus pneumoniae*, or *Pseudomonas aeruginosa* from respiratory samples does not fit the definition of colonization, since it is associated with tissue damage and an inflammatory response [24,25]. Recently, a clinical feature of chronic bronchial infection (CBI) has been suggested to be the appearance of the same PPM in at least three sputum cultures in a year, each separated by at least a month [26]. It is well recognized that there is a strong association between CBI and COPD exacerbations. Most patients with CBI and frequent bacterial exacerbations produce colored/purulent sputum even in the stable state, have more severe dyspnea and an impaired quality of life, and may fulfill radiological criteria for bronchiectasis in chest CTs [24,25,26,27]. Interestingly, a recent prospective study reported that the presence of chronic purulent sputum, number of PPM isolations, and hospitalizations due to the exacerbation of COPD are independent risk factors of bronchiectasis progression in patients with moderate-to-severe COPD [28].

In many previous studies, the regular use of macrolides (e.g., erythromycin, clarithromycin, and azithromycin) has been shown to reduce emergency room visits and hospitalization due to exacerbations of COPD [22,23,24]. The mechanisms of the therapeutic effects of macrolide can go beyond their direct anti-microbial effects; the latest data show that they exert multiple effects on the structure and composition of the lower airway microbiota, with an increased production of bacterial metabolites with anti-inflammatory properties [22,24]. Azithromycin use can reduce the risk of COPD exacerbations, but is also associated with increased incidence of colonization with macrolide-resistant organisms, an excessive rate of hearing decrements, and the prolongation of the QTc interval [23]. In contrast, no changes in the frequency of macrolide-resistant organisms in sputum have been found to appear with the long-term use of erythromycin or clarithromycin [29,30]. Hence, given the increasing prevalence of non-tuberculosis mycobacteria (NTM) diseases, it seems reasonable to consider the use of erythromycin prior to other macrolides. Andrejak et al. [31] reported that the adjusted odds ratio of COPD patients with bronchiectasis for an increased risk of NTM pulmonary disease is 187.5. These data suggest that increased macrolide resistance to NTM can be a significant risk for patients with CBI and frequent bacterial exacerbations.

COPD patients with mucus hypersecretion have a greater loss of lung function and a higher risk of exacerbation [32,33]. Mucolytic agents such as N-acetylcysteine, carbocisteine, or ambroxol may reduce exacerbations and improve health status [33,34]. The European Respiratory Society (ERS)/American Thoracic Society (ATS) guidelines on the management of COPD exacerbations suggested the beneficial effect of mucolytic agents in patients with frequent COPD exacerbations [35].

There is increasing evidence showing that ICS may impair the host defense against pathogenic organisms [36,37], modify the composition of the microbiome, and lead to dysbiosis in COPD patients [38,39]. Current guidelines recommend that ICS may not be indicated in patients with bacterial colonization or recurrent respiratory tract infections [3]. Interestingly, a recent long-term observational study of 201 COPD patients whose airway microbiologies were carefully characterized demonstrated that fewer than 100 circulating eosinophils/μL combined with the presence of CBI may increase the risk of pneumonia in COPD patients treated with ICS [40]. These data support the need for the assessment of airway bacteriology and type 2 airway inflammation in the management of COPD patients, particularly in cases where ICS are deemed necessary.

## 3. Asthma

Airway inflammation in COPD is typically thought to be driven by type 1 immune responses, while type 2 inflammation appears to be present in definite proportions [41,42]. Interestingly, the relationship between COPD and diseases associated with type 2 inflammation from the perspective of impaired lung development is highlighted by many recent epidemiologic studies [43,44,45,46,47,48,49,50]. These studies found that not only personal smoking but also childhood asthma, allergic rhinitis, and eczema were risk factors for reduced pulmonary function and might predict the onset of COPD later in life.

The controversy that asthma and COPD are not always separate diseases has been discussed over the years, and the term asthma-COPD overlap (ACO) has begun to be used. However, no formal definition of ACO has yet been accepted, and the Global Chronic Obstructive Lung Disease (GOLD) 2020 report stated that they no longer refer to ACO, as they constitute two different diseases that may share some common traits and clinical features [3]. Nevertheless, we consider that ACO remains important because of its effects (i.e., poorer quality of life [QOL] [51,52,53], future exacerbations [54,55,56], more rapid decline in lung function [57,58], and higher medical cost [59]) and because it requires personalized COPD management such as interventions with ICS and type 2-directed biologics.

The prevalence of asthma in patients with COPD has varied across studies, based on various biomarkers and cut-off values, including sputum/blood eosinophils, exhaled nitric oxide fraction (FeNO), and IgE/atopy. In a large-scale clinical trial, 20% of patients had blood eosinophilia (≥300 cells/µL) [60]. In a study focused on the FeNO levels in COPD, the prevalence rate of FeNO > 25 ppb was 36.9%, that of >35 ppb was 16.3% [61]. The positive rate of atopy in COPD varies between about 15% and 40% [52,53,61,62,63]. In our study focused on the combination of multiple type 2 biomarkers in ICS-naive COPD patients, more than one-third of the patients had type 2 inflammatory features [64]. Because of the relatively high positive rate, it is essential not to overlook asthma, even in COPD patients who do not have a history of asthma or asthma-like features.

Many researchers have shown an interest in whether type 2 biomarkers can be used to predict the effectiveness of ICS. In particular, many studies have investigated whether blood eosinophils can be used to predict whether patients will benefit from the prevention of future exacerbations by add-on ICS in combination with long-acting β2-agonist (LABA) or LABA/long-acting muscarinic antagonist (LAMA) compared to bronchodilators alone [65,66,67]. Based on these studies, the GOLD report has proposed the use of ICS in combination with bronchodilators in patients with frequent exacerbations and blood eosinophil counts ≥300 cells/μL, while ICS use is not recommended if the blood eosinophil counts are <100 cells/μL [3]. Several studies have reported the usefulness of blood eosinophils to predict the risk of exacerbations after ICS withdrawal from triple therapy (ICS/LABA/LAMA), and only patients with blood eosinophilia (≥300 or 400 cells/μL) were at an increased risk of exacerbations [60,68]. In contrast, some observational studies using add-on ICS did not show an association between blood eosinophilia and a reduced risk of exacerbations [69,70]. This discrepancy may have resulted from influential factors, stability, and the reproducibility of blood eosinophils in patients with COPD [71,72].

**Figure 2:**
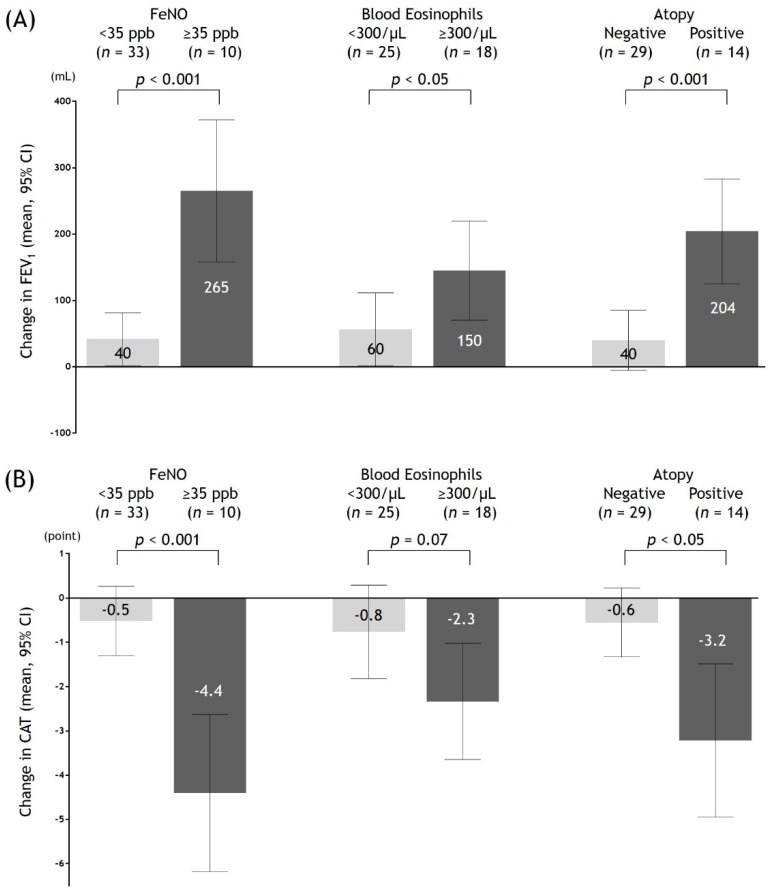
Changes in the mean values (95% CI) of FEV1 (**A**) and CAT (**B**) by 12 weeks of add-on therapy with inhaled corticosteroids, stratified based on the criteria for type 2 inflammations in Japanese guidelines for ACO. Abbreviations: FeNO, exhaled nitric oxide fraction; FEV1, forced expiratory volume in 1s; CAT, chronic obstructive lung disease assessment test; CI, confidence interval; ACO, asthma–COPD overlap.

Recently, we reported a prospective study to detect type 2 inflammation biomarkers for predicting improvements in both symptoms and airflow limitation by ICS in 43 symptomatic patients with COPD who had been taking bronchodilators (de-stress study) [18]. This single-arm study consisted of a 4-week observation period and 12-week treatment period, with add-on inhaled ciclesonide of 400 μg/day. This study excluded current smokers, patients within one month of quitting, and patients with concomitant asthma. An analysis of the effect of ICS on forced expiratory volume in 1 s (FEV1) and symptoms was conducted stratified based on the criteria for type 2 inflammations in the Japanese guidelines for ACO [73]. As indicated in Figure 2, the greater benefit of ICS for patients with higher levels of type 2 biomarkers was demonstrated. Among several type 2 biomarkers, FeNO was identified as the most accurate predictor of improvements in both symptoms and airflow limitation (area under the curve = 0.92).

Because the benefits were small compared with asthma [74,75], no biologics are currently licensed for the treatment of COPD. Whether ACO patients might respond to type 2-directed biologics remains to be fully addressed and requires further investigation.

## 4. Obstructive Sleep Apnea

The coexistence of COPD and obstructive sleep apnea (OSA) has been called overlap syndrome (OVS). OVS is considered to be the result of chance rather than of a pathophysiological link [76,77]. The prevalence of OSA in COPD patients ranges from 9.2% to 28.5% [78]. Patients with combined COPD and OSA have a worse prognosis and an increased risk of cardiovascular events such as hypertension and pulmonary hypertension [79,80]. Since these risks are particularly high in untreated OSA patients, early diagnosis and intervention are required [80]. However, many cases are asymptomatic or not reported separately from the symptoms of COPD itself. Therefore, polysomnography to diagnose OSA is not routinely indicated in COPD patients [81]. Interviewing the bed partner is essential to capture the OSA symptoms.

Management includes patient education to avoid the factors that increase the severity of upper airway obstruction, such as the use of alcohol and hypnotic drugs and weight gain [78,82]. Positive airway pressure is the mainstream treatment for OVS and it has been shown to reduce overall mortality and the risk of COPD exacerbations [82]. However, the efficacy is sometimes limited by patients’ poor adherence. In such cases, nasal high flow may be an alternative means for palliative purposes, but further studies are needed to verify its safety and efficacy [83].

## 5. Pulmonary Hypertension

Pulmonary hypertension (PH), which is one of the significant comorbidities in COPD, is associated with a worse prognosis [84]. Patients with a mean pulmonary artery pressure (mPAP) > 20 mmHg and pulmonary capillary wedge pressure (PCWP) < 15 mmHg with right heart catheterization are defined as PH [85]. The mechanisms of PH-COPD have been considered to be hypoxic pulmonary vasoconstriction, vascular injury, and reduction in the vascular beds, which is due to alveolar destruction by the inhalation of cigarette smoke and harmful substances [86]. Notably, PH-COPD is more prevalent and severe in COPD with pulmonary fibrosis compared to COPD without fibrosis [87,88].

Long-term oxygen therapy suppressed mPAP elevation, and may be effective for PH-COPD with respiratory failure [89,90]. Pulmonary vasodilators which are approved for pulmonary artery hypertension (PAH) may worsen the ventilation/perfusion mismatch and hypoxemia [91,92]. Although the efficacy of PAH-targeted therapy for PH-COPD is still controversial [91,92,93,94], a subgroup of PH-COPD who shows severe pulmonary hypertension despite mild airflow limitation may benefit from pulmonary vasodilators [95,96]. From the perspective of the heterogeneous pathogenesis and poor prognosis of PH patients with COPD, further and different targeted therapies are needed.

## 6. Systemic Comorbidities

### 6.1. Cardiovascular Disease

COPD and cardiovascular disease (CVD) frequently coexist, and the relationship can be explained by shared risk factors, lung hyperinflation, loss of vascular capacity, oxidative stress, and systemic inflammation [97]. The prevalence of CVD in patients with COPD ranges from 20% to 70% [98], with 2.5 times greater odds of CVD in COPD patients compared with the non-COPD cohort [99]. The prevalence of COPD in patients with chronic heart failure (CHF) ranges from 9% to 52% [100], and the presence of COPD and rapid FEV1 decline are risk factors for CHF [101,102]. Arrhythmia is common in COPD patients, and reduced FEV1 is associated with incident atrial fibrillation (AF) [103]. More than 25% of patients with COPD will die as a result of CVD, and 40% of COPD patients with a cardiovascular history will die following a cardiovascular event [104]. There is an increased risk of CVD events, including ischemic heart disease, within the first 30 days after COPD exacerbations in patients with COPD with CVD [105]. It is well known that patients with comorbid COPD and CVD have worse outcomes than those with either condition alone [97]. The presence of CVD is associated with more severe breathlessness and a worse QOL, more frequent hospitalization, and higher mortality than those with COPD alone [104], while the presence of COPD is a predictor of hospitalization and death from cardiovascular events [106].

Despite established associations between COPD and CVD, it is usual for a patient’s presentations to be attributed to one disease only and for the other to be overlooked because of overlapping clinical symptoms and signs. In fact, in studies of COPD patients, excluding those with an existing CHF diagnosis, the prevalence of unrecognized CHF was about 20% [107,108]. Physical examinations such as peripheral edema, systolic murmur, and objective tests such as electrocardiography and the measurement of plasma brain natriuretic peptide can help physicians to identify concomitant CHF in COPD patients [108].

Adequate therapy for CVD is necessary without delay after a new diagnosis of CVD. However, pharmacotherapy for CVD is often withheld due to uncertain beliefs concerning safety for COPD patients. Even when CVD is known, patients with COPD receive less adequate CVD treatment than non-COPD patients [109,110]. In particular, β-blockers are essential medications in CHF, acute myocardial infarction, and AF. The pharmacologic properties of common β-blockers are summarized in Table 1 [111]. A meta-analysis of observational studies reported that β-blocker usage in COPD patients reduces the overall mortality and risk of COPD exacerbations (28% and 38%, respectively) [20]. Importantly, selective and non-selective β-blockers have different outcomes for patients with COPD. A previous randomized study that compared the effect of bisoprolol and carvedilol in patients with concurrent COPD and CHF confirmed that bisoprolol improved FEV1 and caused fewer adverse events than carvedilol [112]. In a retrospective study, patients taking bisoprolol were at a lower risk of CHF and/or COPD exacerbation than patients taking carvedilol [113]. In another cohort study, the rate of mortality and CHF exacerbations was lower in patients treated with bisoprolol compared to those with carvedilol or metoprolol [114]. Very recently, in a large-scale randomized controlled trial of metoprolol for patients with COPD that specifically excluded patients with established indications for a β-blocker, the metoprolol group did not show a lower risk of COPD exacerbations than the placebo group [115]. From the evidence so far, COPD patients without any cardiovascular comorbidity do not require beta-blockers, while the selective β-blocker bisoprolol should be used in COPD patients with CVD.

Recently, the CLAIM study showed that dual bronchodilator treatment in hyperinflated patients with COPD reversed the detrimental lung-heart imbalance by increasing pulmonary microvascular blood flow, left ventricular end-diastolic volume, stroke volume, and regional ventilation [116,117]. These data clearly highlight the multidimensional benefits of maximum bronchodilation in patients with COPD.

### 6.2. Malnutrition/Obesity

The association between low body mass index (BMI) and poor prognosis in patients with COPD independently of the ventilation impairment is well known [118,119]. The prevalence of loss in COPD is associated with the presence of emphysema [120]. Body weight loss is particularly prevalent in patients with severe COPD and chronic respiratory failure, occurring in ~50% of such patients, but can be also seen in 10–15% of patients with mild-to-moderate COPD [121].

Loss of muscle mass is the major cause of the weight loss, whereas the loss of fat mass contributes to a lesser extent [121]. The assessment of the nutrition status and targeted intervention for specific metabolic types are essential. The measurements of body composition, including the distribution of fat-free mass, fat mass, bone mass, and density, needs to be assessed to obtain information concerning variables related to the disease severity and exercise capacity in patients with COPD [122]. Schols et al. showed that the prognosis improved in COPD patients if body weight could be regained after nutritional support, despite the absence of improvements in lung function [123].

Obesity paradox is a phenomenon in which obese patients with COPD survive longer than patients who are not obese, despite the cardiovascular risk and inflammatory burdens associated with obesity [124,125]. Although the detailed mechanism is unclear, Ji et al. recently reported that a low BMI is associated with poorer survival, but not for the risk of exacerbations or pneumonia, or for the need for medical care in hospital [125].

### 6.3. Gastroesophageal Reflux

An association between gastroesophageal reflux (GER) and COPD has been recognized. The prevalence of GER in COPD patients is higher than that in the non-COPD population, ranging from 19% to 78% [126,127,128]. COPD patients with GER are at greater risk of COPD exacerbation [129,130] and are associated with worse symptoms [131]. The underlying mechanism of GER in COPD patients has been considered to be micro-aspiration and bronchoconstriction due to vagal nerve reflex induced by esophageal acid reflux, while GER also causes inflammation and edema in the airways, increases bronchial hyperresponsiveness [132,133]. Additionally, lung hyperinflation may be associated with lower esophageal sphincter (LES) relaxation, which can lead to worsening of the acid reflux [134].

Previous studies showed that the administration of proton pump inhibitors (PPIs) in COPD patients with GER improved symptoms and reduced COPD exacerbations [127,135], but another study did not [136]. It is still controversial whether anti-reflux therapy is effective for COPD patients with GER.

### 6.4. Anxiety/Depression

Depression is more prevalent in COPD patients than in a comparable general cohort [137,138]. A recent meta-analysis reported approximately 30% of the COPD group and 10% of the control group had a depressive status [139]. Anxiety tended to be similar, and the prevalence was higher in patients with more severe COPD [137,138,139]. Depression may be primarily driven by a patient’s perception of a serious chronic disease comprising symptoms and limitations to daily activities rather than by the underlying inflammatory pathology in COPD [137]. Many recent studies have shown that depression is associated with a worse quality of life, poor adherence to treatment plans, exacerbations, increased hospitalizations, and more health care costs in patients with COPD [130,140,141].

COPD patients with anxiety and/or depression symptoms will report higher COPD Assessment Test (CAT) scores compared to those without symptoms [142]. In COPD patients with high CAT scores despite adequate COPD treatment, the use of a psychological screening test such as the Hospital Anxiety and Depression Scale (HADs) may be useful for the diagnosis of depression and anxiety [143]. Comprehensive pulmonary rehabilitation programs with exercise therapy, relaxation techniques, and self-management education appeared to be helpful in reducing depression and anxiety [138,143]. By contrast, a recent meta-analysis could not provide conclusive treatment recommendations for the use of antidepressants in patients with COPD [144]. There remain unanswered questions about adequate treatment strategies for comorbid anxiety/depression in patients with COPD.

### 6.5. Sarcopenia/Skeletal Muscle Dysfunction

Sarcopenia is one of the representative extrapulmonary manifestations in COPD patients [145], and its prevalence is reported to be 15–25% [146,147]. Sarcopenia is characterized by a loss of skeletal muscle mass/function and is categorized as secondary (e.g., disease) or primary (e.g., aging, disuse) [148]. Currently, several methods evaluating low muscle mass and function based on the features of sarcopenia are commonly used as criteria for its diagnosis [149,150]. Aging usually cause a shift from type 2 (fast-twitch muscle fiber) to type 1 myofibers, which have a high density of mitochondria in muscle cells (slow-twitch muscle fiber). However, there is a transition from type 1 to type 2 myofibers in COPD [151]. This structural alteration as well as quantitative reduction in muscle which is related to aging could contribute to COPD sarcopenia. Additionally, inflammation, oxidative/nitrative stress, mitochondrial dysfunction, motor neuron loss, microvascular changes, imbalance in protein metabolism, and apoptosis of muscle cells may be involved in the underlying mechanisms in the pathophysiology of COPD sarcopenia [148,151,152,153].

The associations between COPD and sarcopenia may be bidirectional, because sarcopenia could not only cause COPD progression but also follow the disuse of muscle accompanied with the progression [145,146]. Theoretically, we should consider treatment for both sarcopenia and COPD itself. When focusing on sarcopenia, pulmonary rehabilitation including intensive training can reverse sarcopenia in some patients with COPD [146]. Since the reduction in slow-twitch type 1 myofibers offering endurance capacity are observed in COPD, the sustainable strengthening of low-intensity exercise such as daily living activity could be an effective approach. Additionally, a combination of rehabilitation and another therapeutic strategy such as adequate nutritional support might be useful to improve sarcopenic obesity and cachexia [154].

### 6.6. Frailty/Sedentary Lifestyle

Frailty is defined as a clinical state of vulnerability to stressors following age-associated deterioration in multiple organs and molecular systems [155]. The prevalence range of frailty in COPD is 20–57% [156,157]. The physical characteristics include muscle weakness, weight loss, low energy production, and reduced exercise tolerance [156,157]. Frailty often leads to the onset of a negative spiral called the “cycle of frailty”, and it is significantly associated with adverse health outcomes such as sedentary lifestyle, hospitalization, and mortality [155,156,158,159]. In fact, Vaz Fragoso et al. reported that frail patients who also had respiratory impairment had a nearly 4-fold increased risk of death relative to those who were non-frail and had normal pulmonary function [158]. Furthermore, patients with COPD and frailty are more likely to be readmitted within 90 days of hospitalization for COPD exacerbations, even after adjusting for age and several disease-related factors [159].

Of importance, frailty could be reversible because it is a dynamic process with frequent transitions between frailty status over time [160]. Actually, rehabilitation programs improve the frail status, the frailty could be changed for approximately one third of patients with stable COPD, and the transitions are correlated with meaningful changes in clinical outcome [161,162]. Moreover, a recent prospective study of COPD patients with an admission history reported that a greater quantity of low-intensity physical activity reduces the risk of hospitalization due to COPD, but high-intensity physical activity does not produce any risk reduction [163].

Many previous studies have shown that most patients with COPD spend significantly less time walking and standing and more time sitting and lying in their daily life when compared with sedentary healthy elderly subjects [164,165,166]. Recently, dual bronchodilator therapy has been shown to reduce the sedentary time by 8.64 min in COPD patients [167], but it remains unclear whether this indicates a clinical improvement with drug treatment and whether this improvement can contribute to better clinical outcomes. In patients with COPD, reduced physical activity is thought to have a behavioral component, in which patients choose to reduce their activity. Self-limiting activities to reduce dyspnea can give the impression that the patient is less symptomatic than they actually are, but it is important that patients know that activity avoidance can exacerbate dyspnea [10]. Therefore, it might be ideal to focus on behavior change, targeting low-intensity activity such as daily physical activity in order to improve frailty and sedentary behavior. In fact, a self-management behavior- modification program combined with exercise training and bronchodilation has been shown to be an effective intervention to reduce physical activity-related dyspnea and difficulty [168]. Moreover, a recent prospective study reported that the combination of the assist use of inhaled short-acting beta-2 agonists (SABA) and coaching targeting self-limiting daily activities significantly improved physical activity in COPD patients [169]. In this report, the physicians initially identified patient-specific restrictions of daily behavior due to dyspnea, then coached patients to conduct the adequate use of SABA prior to their limited physical activity [169]. These data suggest that the combination of a bronchodilator with coaching based on self-limiting activities may be a useful approach to modify the sedentary lifestyle of COPD patients.

**Figure 3:**
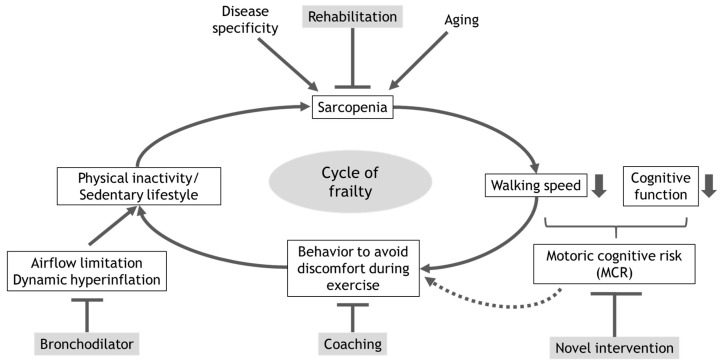
The schematic view of the vicious cycle of frailty/sedentary lifestyle in patients with COPD and potential approaches to attenuate the negative impact of this cycle.

The coexistence of immobility and mild cognitive impairment could be an early sign of dementia, and this status is currently proposed as motoric cognitive risk (MCR) [170,171,172]. Since dementia has a direct impact on quality of life and social activities for COPD patients, clarifying the association between frailty and MCR will help to develop a novel approach to avoid a sedentary life and cognitive risk. The view of the vicious cycle of frailty/sedentary lifestyle in patients with COPD and potential approaches to attenuate the negative impact of this cycle are summarized in Figure 3.

## 7. Future Directions

By reviewing the current literature, several promising approaches can be identified for comorbid conditions in COPD patients, such as bronchiectasis, asthma, heart failure, sleep apnea, malnutrition, and frailty. Importantly, the published evidence shows that these interventions bring significant improvements in patient-centered outcomes, including symptoms, dyspnea, exacerbations, and quality of life. By contrast, there remain unanswered questions about adequate treatment strategies for comorbid pulmonary hypertension, gastroesophageal reflux, anxiety, and depression. Nevertheless, several challenges can also be found in the new treatment strategy that proposes to identify treatable conditions early and to treat patient-specific clinical characteristics in parallel with usual care [9]. This approach must be adapted to the situation of clinical practice, taking into account the characteristics of the patients. However, medical resources vary greatly from country to country. In some regions, it is difficult to access some diagnostic tools, such as CT, FeNO, echocardiography, and polysomnography, and some therapeutic agents may not be approved. The low utilization of these resources can cause an underestimation of comorbidities and is a significant barrier to adequate disease management. Moreover, since COPD is a very common disease, it is ideal that the multiple comorbid conditions can be comprehensively and easily assessed by primary care providers, pulmonary specialists, and other physicians. If the evaluation method is highly accurate but requires expert knowledge and skills, it will not be fully used, and, as a result, treatable comorbidities will be latent in COPD patients without proper management. From this viewpoint, it is important to develop simplified diagnostic criteria and indices of disease activity. Very recently, some emerging statements on how to assess the coexistence of asthma characteristics in patients with COPD have started to provide simplified diagnostic criteria including symptoms, personal history, and type 2 inflammation biomarkers [73,173,174]. Henceforth, similar approaches have the potential to promote personalized COPD management for challenging patients with remaining unmet needs.

## Figures and Tables

**Table 1:** Pharmacological properties of β-blockers.

	Metoprolol	Carvedilol	Bisoprolol
Daily oral dose *	12.5–200 mg, QD	3.125–100 mg, BID	1.25–10 mg, QD
Plasma t^1/2^	3–7 h	6–10 h	10–12 h
Selectivity ratio (β1:β2)	20:1	NA	75:1
α-antagonism	No	Yes	No
Lipid solubility	High	Moderate	Moderate
Bioavailability	50%	30%	>90%
Clearance	Liver	Liver	Liver/Kidney
Metabolism	CYP2D6	CYP2D6	CYP2D6

* Dosage for chronic heart failure. Abbreviations: QD, once a day; BID, twice a day; t^1/2^, half-time; NA, not available; CYP2D6, cytochrome P450 2D6.
